# Trends in the prevalence of smoking in Portugal: a systematic review

**DOI:** 10.1186/1471-2458-12-958

**Published:** 2012-11-08

**Authors:** Helena Carreira, Marta Pereira, Ana Azevedo, Nuno Lunet

**Affiliations:** 1 Department of Clinical Epidemiology, Predictive Medicine and Public Health, University of Porto Medical School, Al. Prof. Hernâni Monteiro, Porto, 4200-319, Portugal; 2 Institute of Public Health of the University of Porto, Rua das Taipas, nº 135, Porto, 4050-600, Portugal

**Keywords:** Epidemiology, Portugal, Smoking, Trends

## Abstract

**Background:**

Understanding the dynamics of smoking at the population level is essential for the planning and evaluation of prevention and control measures. We aimed to describe trends in the prevalence of smoking in Portuguese adults by sex, age-group and birth cohort.

**Methods:**

PubMed was searched from inception up to 2011. Linear regression was used to assess differences in prevalence estimates according to the type of population sampled, and to estimate time trends of smoking prevalence considering only the results of studies on nationally representative samples of the general population.

**Results:**

Thirty eligible studies were identified. There were statistically significant differences in the prevalence estimates according to the types of population sampled in the original studies. Between 1987 and 2008, the prevalence of smoking increased significantly among women aged ≤ 70 years; the steepest increase was observed in those aged 31–50 and 51–70 years (from 4.6% and 0.1% in 1988, respectively, to 16.4% and 4.5% in 2008, respectively). The prevalence of smoking increased in all birth cohorts, except for those born before 1926. In the same period, among men, smoking decreased in all age-groups, with steepest declines in those aged ≤ 30 years (from 41.8% in 1988 to 28.8% in 2008) and those aged ≥ 71 years (from 15.1% in 1988 to 4.6% in 2008). The prevalence of smoking declined among men of all birth cohorts.

**Conclusions:**

This study provides robust evidence to place Portuguese women at stage II and men at the later stages of the tobacco epidemic.

## Background

Smoking is the main preventable cause of premature death [1]. In 2004, it caused nearly 5 million deaths worldwide, accounting for approximately 25% and 7% of the mortality among adult European men and women, respectively [2]. In Portugal, in 2005, it was estimated that 18% of the deaths in men and 5% in women were attributable to smoking, accounting for more than 70 thousand disability-adjusted life years [3].

The most comprehensive data on smoking in Portugal comes from the four National Health Surveys [4-7] and from several Eurobarometer surveys [8-14]. However, most of the latter did not provide sex- and age-specific estimates, which are essential to plan, monitor and evaluate the impact of prevention and control measures. Other studies assessed smoking behaviours in different calendar years, mainly from smaller regional samples recruited across the country, but the usefulness of these estimates has not been systematically addressed before.

A general model for the tobacco epidemic is widely accepted [15], though not all countries followed the same pattern as the ones used to formulate this model a few decades ago [16]. Country-specific data on the patterns of smoking at a population level are, therefore, necessary to understand its dynamics in each setting.

We conducted a comprehensive systematic review of studies that quantified the prevalence of smoking in Portuguese adults. Our specific aims were to compare the estimates obtained from studies that evaluated populations with different characteristics and to estimate the trends in the prevalence of smoking in Portugal, by sex, age-group and birth cohort.

## Methods

### Search strategy

We searched PubMed from inception up to January 2011, to identify original studies and review articles addressing the distribution of smoking in samples of the Portuguese adult population. The detailed search expression is presented in the systematic review flowchart (Additional file 1: Figure S1). This is a comprehensive search expression that includes terms directly related with smoking behaviours (“smoking”, “smoke”, “tobacco”, “cigarette”), as well as with other cardiovascular risk factors (hypertension, obesity, dyslipidaemia, diabetes, physical inactivity). The latter were also included because smoking is frequently associated with other behaviours with potential impact in human health [17,18], and therefore reports with primary objectives not directly related with the assessment of smoking behaviours may also provide data of interest for this review, as secondary results. The reference lists of the review articles addressing the distribution of cardiovascular risk factors were screened to identify potentially eligible original reports. Additionally, we searched for reports that are by nature not suitable for indexation in journal databases (e.g. Eurobarometer study).

### Eligibility criteria and screening of reference lists

Two reviewers independently evaluated the studies in three consecutive steps, following predefined criteria, to determine the eligibility of each report. The first two steps relied on the same criteria. In step 1, the exclusion of irrelevant studies was decided by considering only the title and abstract; when the abstract of a particular article was not available, the article was selected for evaluation in step 2, except when the title unequivocally presented information for exclusion (e.g. case report, studies of risk factors in a specified population). The full text of studies selected for step 2 were then evaluated to decide on their eligibility. The studies selected for step 3 were re-evaluated to determine their adequacy for extraction of relevant data.

The criteria for exclusion of studies were the following: reports not written in Portuguese, English, Spanish, French or Italian; studies not involving humans (e.g. *in vitro* or animal research); editorials, reviews or comments; reports not providing data specifically for Portuguese subjects; studies not evaluating adult populations; studies in which sample selection was dependent of at least one cardiovascular risk factor (hypertension, obesity, dyslipidaemia, diabetes or physical inactivity) and therefore participants could not represent the general population regarding the prevalence of smoking (e.g. subjects with diabetes, athletes, sedentary elderly); insufficient characterization of the methods (e.g. not specifying the region where the sample was assembled); not presenting sex-specific data on smoking. We did not exclude from the systematic review the studies presenting data not stratified by age, although these were not eligible for all data synthesis.

When more than one report referred to the same study, we considered the one providing data for the largest sample or, when the sample was the same, we used the source presenting the results with more detail, although any of these reports could be used to obtain information on the study characteristics.

The disagreements between the independent assessments of the reviewers were resolved by consensus or after discussion with a third researcher.

### Data extraction

Two investigators independently evaluated the selected studies to extract the following data for sample characterization: sex; age; sample size; type of population (general population, university students, occupational groups, users of primary health care centers or volunteers); sampling strategy (probability or non probability sampling); geographical coverage (national or regional).

We considered the study population to be general population when subjects were randomly selected from the electoral rolls or recruited from the registries of the primary health care centers. The latter were considered general population because in Portugal the access to the National Health Service is universal and in theory everyone is registered, including those who do not use it as the main source of health care. The studies that evaluated samples of the general population were further divided according to the geographical coverage of the study, into regional or national representative samples. University students, occupational groups, users of primary health care centers (when subjects were recruited among the attendants to health care centers or data were abstracted from clinical records) and volunteers (when subjects took some initiative to participate in the survey) were considered different types of population.

We extracted age-specific prevalence estimates of current smoking, whenever available. Due to the large heterogeneity in the criteria to define classes of smoking, we considered that the data referred to current smoking when described in the original reports as: “currently smoking”; “currently smoking or had ceased for less than one year”; “smoking cigarettes but not as many as one per day, or smoking more than one cigarette per day, or smoking pipe or cigars”; “regular smoking”; “smoking daily”; “smoking daily for at least six months”.

The mean age of each age-group was extracted, whenever available. For the studies that did not present the mean age of the participants in each age-group we assumed the mid-point of the age interval; for the open age intervals at the extremes we estimated the mid-point by adding and subtracting the width of the closest class to the upper and to the lower limits, respectively (e.g. for surveys reporting data in participants aged < 30, 30–39, 40–49, and ≥ 50 years, we considered the overall range as 20–59 years). When an age-group also included subjects aged below 18 years old (e.g. 17–20 years), we computed the mid-point and excluded the data if the mid-point year was lower than 17.5 years old.

We obtained age- and sex-specific estimates directly from the authors of three studies [7,19,20], including one of the largest regional surveys and two national surveys.

Differences in the data extracted by the two investigators were discussed until consensus, and involving a third investigator whenever necessary.

### Data analysis

We compared the estimates of current smoking across the different types of population, considering national representative samples of the general population as the reference class. To quantify these differences, we fitted sex-specific multiple linear regression models, adjusting for the mean age of the participants (continuous variable), the year of survey (continuous variable), the smoking measure involved (daily smoking/current smoking, categorical variable) and geographical coverage of the study (national/regional, categorical variable). Since more than one estimate of the smoking prevalence could be extracted from each report, corresponding to different age strata, the confidence intervals derived from the multiple linear regression models were calculated using robust estimates of the standard errors [21], to account for the dependence between the observations from the same study.

To estimate the time trends of the prevalence of smoking, we considered only the results of the studies that evaluated samples with national coverage that were representative of the general population [14,20,22-24]. We conducted sex-specific stratified analyses for four age-groups and according to birth cohorts. We selected the age-groups ≤ 30, 31–50, 51–70 and > 70 to represent young, young middle aged, middle aged and older subjects.

As some of the studies included in the review evaluated samples of subjects in a wide range of ages, and therefore the estimated mid-point of the age-group may not correspond to an accurate estimation of the age of a large proportion of the subjects evaluated, we excluded the results of these studies from the time trends analyses [14,24].

To compute the year of birth of the participants, we subtracted the mid-point age of each age-group from the calendar year of the survey. This variable was further categorized using the quartiles of the distribution as cutoffs: ≤ 1926; 1927–1946; 1947–1959; ≥ 1960.

The results are presented in figures, describing the sex-specific variation of the smoking prevalence over time for each age group and birth cohort. We included in the figures only one estimate per age-group from each study. When studies reported estimates by strata of age that were narrower than the age-agroups we defined for analysis, it corresponded to the average of the prevalence in the narrower age strata, weighted by the corresponding number of participants. Each figure also includes a line representing the linear or quadratic (whenever significantly different from the linear) prediction of the prevalence of current smoking as a function of the year of survey.

We also provide estimates of the prevalence of smoking obtained from the linear regression models in selected calendar years, for each age-group and birth cohort.

## Results

### Systematic review

Thirty studies were eligible for the systematic review. Detailed information on their main characteristics, as well as the respective prevalence estimates, are provided in the Additional file 1: Table S1. The reports were published between 1990 and 2012, and referred to data collected between 1987 and 2012.

Estimates of current smoking in women and in men were available from 26 and 28 reports, respectively, 8 of which evaluated mainland/national samples. The studies were conducted in 5 different types of population, including general population (n = 14), occupational groups (n = 3), university students (n = 6), users of primary health care centers (n = 6) and volunteers (n = 1) (Additional file 1: Table S1).

### Estimates of the prevalence of current smoking according to the type of population

**Figure 1:**
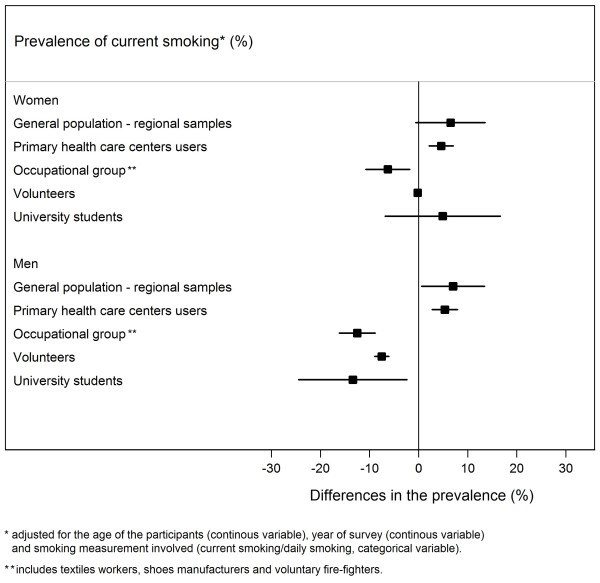
**Differences in the estimates of the prevalence of current smoking according to the type of population sampled, in comparison with samples of the general population with national representativeness, in women and men. **The prevalences were estimated using sex-specific models including the prevalence of smoking as dependent variable and the type of population, the mean age of participants (continuous variable), the year of survey (continuous variable), the geographical coverage of the study (national/regional, categorical variable) and the smoking measurement (current smoking/daily smoking, categorical variable) of the study as independent variables.

Having as reference the results from the 6 studies that evaluated national samples of the general population and provided age-stratified estimates, the prevalence of current smoking among women, adjusted for the age of the participants, year of survey and measure of smoking involved, was significantly higher in primary health care users [4.6%; 95% confidence interval (95% CI): 2.1 to 7.0]. Among men, the prevalence of current smoking was significantly higher in regional samples of the general population (7.0%; 95% CI: 0.1 to 13.4) and primary health care centres users (5.3%; 95% CI: 2.7 to 7.9); it was significantly lower in samples of specific occupational groups (−12.5%; 95% CI: -16.2 to −8.8), volunteers (−7.5%; 95% CI: -8.9 to −6.1) and university students (−13.4%; 95% CI: -24.5 to −2.4) independently of the age of the participants, survey year and measure involved to define smoking (Figure 1).

### Trends in smoking prevalence in the general population

**Figure 2:**
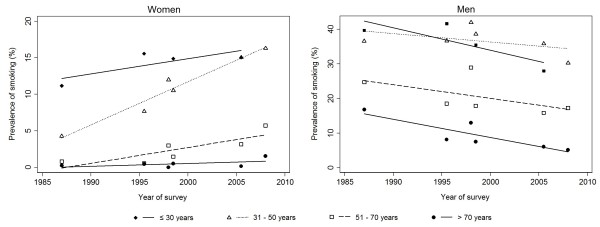
**Trends in the prevalence of smoking in different age group, by sex. **Only were considered the results of the studies involving national representative samples of the general population. When a given study provided more than one estimate for each of the age groups computed, we computed the weighted mean of the prevalence of smoking. Studies involving samples with a wide age-range and not presenting age-stratified data were excluded.

**Figure 3:**
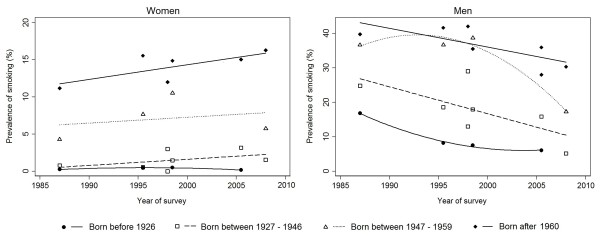
Trends in the prevalence of smoking in different birth cohorts, by sex.

Between 1988 and 2008, the prevalence of current smoking increased significantly among women of all ages, except in those older than 70 years (Figure 2). The steepest increase was observed in those aged 31–50 years and 51–70 years (from 4.6% and 0.1% in 1988, respectively, to 16.4% and 4.5% in 2008, respectively); a less pronounced upward trend was reported in younger women (Figure 2 and Table 1). The prevalence of smoking increased in all cohorts, except in women born before 1926, though the absolute variation was much smaller than the observed in the analysis by age-group (Figure 3 and Table 1).

**Table 1:** Estimated prevalence of smoking by age group and birth cohort

	Estimated prevalence of smoking (%) and 95% confidence intervals *					
	**Women**			**Men**		
	**1988†**	**1998†**	**2008†**	**1988†**	**1998†**	**2008†**
Age group						
≤ 30 years	12.4 (8. 3 to 16.5)	14.4 (12.0 to 16.9)	15.9 (12.4 to 19.3)	41.8 (30.6 to 52.9)	35.3 (28.7 to 41.9)	28.8 (18.0 to 39.7)
31 – 50 years	4.6 (3.0 to 6.3)	10.5 (9.4 to 11.6)	16.4 (15.7 to 17.2)	39.3 (32.2 to 46.4)	36.9 (32.9 to 40.8)	34.4 (27.4 to 41.5)
51 – 70 years	0.1 (0.0 to 2.4)	2.3 (1.0 to 3.5)	4.5 (2.2 to 6.7)	24.8 (18.7 to 30.9)	20.9 (15.9 to 25.9)	16.9 (12.2 to 21.6)
≥ 71 years	0.1 (0.0 to 0.7)	0.5 (0.2 to 1.0)	0.9 (0.0 to 2.1)	15.1 (10.9 to 19.3)	9.9 (7.2 to 12.6)	4.6 (2.4 to 6.8)
Birth cohort						
≤ 1926	0.3 (0.0 to 1.0)	0.5 (0.1 to 1.0)	0.0 (0.0 to 1.2)	15.5 (8.5 to 22.5)	7.3 (1.5 to 13.1)	6.8 (0.0 to 19.3)
1927 – 1946	0.6 (0.0 to 2.8)	1.5 (0.3 to 2.6)	2.3 (0.3 to 4.3)	26.1 (14.3 to 37.8)	18.3 (12.3 to 24.3)	10.5 (0.0 to 21.2)
1947 – 1959	6.3 (0.0 to 17.3)	7.1 (0.2 to 14.0)	7.9 (0.0 to 19.9)	37.3 (3.1 to 71.4)	37.0 (8.3 to 65.8)	17.5 (0.0 to 57.0)
≥ 1960	12.0 (9.3 to 14.7)	13.9 (12.5 to 15.3)	15.9 (13.8 to 18.1)	42.5 (34.6 to 50.4)	37.1 (33.0 to 41.1)	31.6 (25.4 to 37.9)

* Prevalence estimated from sex-specific linear regression model, for each age group and birth cohort;

† The years 1988 and 2008 were selected because correspond approximately to the first year and last years with available data, respectively, and 1998 because is the mid-point of this period.

In the same 20 year-period, the prevalence of smoking among men decreased in all age-groups, with steepest declines observed among those aged ≤ 30 years (from 41.8% in 1988 to 28.8% in 2008) and those aged ≥ 71 years (from 15.1% in 1988 to 4.6% in 2008) (Figure 2 and Table 1). The prevalence of smoking has declined linearly among men born between 1927 and 1946, and in those born after 1960. For the cohorts of men born before 1926, the prevalence decreased, mainly before 1995, and stabilized thereafter, while among those born between 1947 and 1959 a steep decline was observed only in the second half of the period of analysis (Figure 3).

## Discussion

This study shows that in the last decades the smoking prevalence increased markedly among Portuguese women, while it decreased among men. The results from the studies involving samples of the general population without national representativeness overestimate the national prevalence of smoking, showing that these studies are of limited interest when the purpose is to estimate time trends in the general population.

Smoking is strongly associated with morbidity, and the lower prevalence observed in the samples of specific occupational groups may result from selection bias, as subjects need to be healthy enough to be active workers and those who develop diseases are more likely to leave their employment [25]. Among workers, smoking has also been associated with greater absenteeism and injuries [26,27], which contributes to a lower probability of smokers being selected for the epidemiological investigations.

In the only study whose participants we considered volunteers, the healthy volunteer effect is likely to have occurred, as those who have a proactive attitude to participate in a survey tend to be more sensitive to health messages than the average person from the general population [28,29].

Primary health care users had significantly higher levels of smoking, both in women and in men. Smoking is strongly associated with a higher incidence of several chronic diseases [30,31] and the prevalence estimates for this group may reflect the increased use of health care services by smokers.

The gender differences in the prevalence of smoking among university students are likely to be explained by Portuguese women being at earlier stages of the smoking epidemic than men.

According to the most recent update of the smoking epidemic model [16], among women the stage II is characterized by a rapid increase of the prevalence of smoking, along with few deaths attributable to smoking; stage III starts when the peak prevalence is achieved. Our results on the trends in the prevalence of smoking place Portuguese women in the second stage of the epidemic [15,16]. However, between 1955 and 2005, the lung cancer mortality rates increased 1.6% (95% CI: 1.4% – 1.8%) per year among Portuguese women (35–74 years) [32], which is compatible with both the end of stage II and stage III of the smoking epidemic.

The data referring to men suggest an ongoing transition from the third to the fourth phase of the smoking epidemic. In phase III, the prevalence of smoking begins to decline, while the smoking-attributable mortality rises rapidly [15,16]. In phase IV, the prevalence continues to decline while the mortality attributable to smoking peaks in the beginning of this stage and declines thereafter [15,16]. Among Portuguese men aged 35 to 74 years, between 1986 and 1996, the annual percent change (APC) in the lung cancer mortality rate was 1.52% (95% CI: 0.59% to 2.46%), and between 1996 and 2005 the rates stabilized (APC: -0.15%, 95% CI: -0.99% to 0.69%) [32].

Previous studies involving cross-sectional analyses have placed Portugal at earlier stages of the epidemic than most European countries [23,33]; Spain, the neighbour country, is at the beginning of stage IV [34]. We put forward a sex-specific classification for the Portuguese population, in accordance with the recent review of the smoking epidemic model [16]. The earlier stage proposed for women might be a consequence of Portugal’s lower economic and social development, which may have determined a slower spread of the epidemic among the less educated women who are particularly affected by prices increases [35,36]. Portugal may take advantage of this position among women, by implementing effective public health measures to curb the trends observed and to avoid the harmful effects of smoking in the next decades.

The present review is based on an extensive literature search and provides a comprehensive summary of the best available evidence on the prevalence of smoking in Portugal. However, there are some limitations that need to be addressed.

The studies included in the systematic review are heterogeneous in what concerns the criteria to define smoking, involving measures of current and daily smoking, possibly contributing to an underestimation of the smoking prevalence and trends in Portugal. However, there were no statistically significant differences according to the criteria involved and a sensitivity analysis that excluded the studies only providing data on daily smoking yielded virtually the same results.

Another limitation of this study results from the inclusion of samples of subjects aged above 18 years, while the smoking behaviours of the population aged above 15 years are important for a more comprehensive understanding of the dynamics of smoking and the tobacco epidemic.

Portugal signed the World Health Organization Framework Convention on Tobacco Control and already adopted measures to control tobacco consumption. These include health warnings in the cigarette packages, tax increases and, more recently, a law regulating the use of tobacco in restaurants and other public places, aiming to protect subjects from passive smoking exposure [37]. The strict enforcement of the latter measure occurred in 2008 and may have had a greater impact on the smoking behaviours of the population. However, we only obtained age-stratified data on the smoking prevalence until 2008 and therefore the impact of this measure could not be ascertained in the present study.

After 1998, the smoking prevalence has been declining among all birth cohorts of men. This is in accordance with the decreasing sales of tobacco products in Portugal, which were estimated to be nearly 18 billion cigarettes in 2002, and less than 12 billion in 2011 [38]. Notwithstanding, the trends reported in this study, especially among women, demand for the reinforcement of the existing policies and for more effective policies targeting the whole population. In Portugal the percentage of per capita gross domestic product needed to purchase 100 packs of cigarettes was 1.66 in 1990 and 1.76 in 2006, representing a small change in the cigarettes affordability in the country over the last decades [39]. Price increases have been associated with reduced consumption and quitting smoking [40], and tax increases may be particularly important in the current local scenario of economic and financial crisis. Furthermore, they have the potential to affect particularly lower socio-economic groups, among whom the prevalence of smoking is higher in men [23] and expected to increase among women [19]. Other measures, such as the promotion of the smoking cessation clinics already existing in the country and the creation of new ones, as well as the free provision of nicotine-replacement therapies and other similar aids, may contribute to downward trends [41]. The monitoring of the epidemic should also consider the study of other tobacco products expected to be increasingly used with the rising prices and decreasing purchasing power [40].

## Conclusions

Our results place Portuguese women in the stage II of the smoking epidemic, while men are at the later stages, between stages III and IV.

## Competing interests

The authors declare that they have no competing interests.

## Authors’ contributions

HC collaborated in the acquisition, analysis and interpretation of the data, and wrote the first draft of the article. MP collaborated in the design of the study, data collection and revision of the article. AA and NL designed the study, analyzed and interpreted the data, and reviewed the article critically for important intellectual content. All authors read and approved the final manuscript.

## Pre-publication history

The pre-publication history for this paper can be accessed here:

http://www.biomedcentral.com/1471-2458/12/958/prepub

## Supplementary Material

Additional file 1 **Figure S1. **Systematic review flowchart. The studies identified through PubMed search and screening of the bibliographic references of the review articles were evaluated independently by two researchers, in three consecutive steps, following pre-defined criteria. Thirty studies, from five distinct type of population, were eligible for systematic review. **Table S1 **Main characteristics and results of the studies included in the systematic review and respective prevalence estimates. Click here for file
