# Supplementary material for: Trends in the prevalence of smoking in Portugal: a systematic review
Source: BMC Public Health. 2012 Nov 8;12:958. doi: 10.1186/1471-2458-12-958 (PMC3544737; doi:10.1186/1471-2458-12-958)
Supplement: Additional file 1 — Figure S1. Systematic review flowchart. The studies identified through PubMed search and screening of the bibliographic references of the review articles were evaluated independently by two researchers, in three consecutive steps, following pre-defined criteria. Thirty studies, from five distinct type of population, were eligible for systematic review. Table S1 Main characteristics and results of the studies included in the systematic review and respective prevalence estimates. [file 1471-2458-12-958-S1.docx]

**Additional file 1.** Systematic review flowchart.

**2958 publications**

(2887 identified through Pubmed search and 71 from bibliographic references of reviews)

Search expression:

[humans[MeSH Terms] AND (Portugal[ad] OR portugal OR acta med port OR rev port cardiol OR rev port cir cardiotorac vasc OR rev port pneumol OR acta reumatol port OR lisboa[ad] OR lisbon[ad] OR (porto[ad] NOT (brasil[ad] OR brazil[ad])) OR coimbra[ad] OR braga[ad] OR covilha[ad]) AND ((hypertension OR "high blood pressure" OR "blood pressure" OR systolic OR diastolic) OR (obes* OR "body mass index" OR bmi OR overweight) OR (cholesterol OR triglycerides OR HDL OR LDL OR dyslipidemia) OR (**smoking OR smoke OR tobacco OR cigarette**) OR (diabetes OR glycemia OR hyperglycemia OR "impaired fasting glucose" OR IFG OR "impaired glucose tolerance") OR ("physical activity" OR "leisure activities" OR motor activity[mh] OR sedentariness OR exercise))]

**1892 studies excluded based on title and abstract assessment:**

10 non eligible language

156 non humans

254 case reports

393 reviews or editorials

53 non Portuguese subjects

178 non adult population

848 sample selection dependent on cardiovascular risk factors

**1066 publications**

**822 studies excluded based on full text assessment:**

52 non humans

17 case reports

211 reviews or editorials

47 non Portuguese subjects

24 non adult population

274 sample selection dependent on cardiovascular risk factors

176 no data about risk factors or lack of information

21 data already described

**244 publications**

**156 studies excluded during data extraction process:**

34 reviews or editorials

32 not presenting data in an eligible format

59 data already described

31 insufficient information to characterize population

**88 publications**

10 studies* with data on smoking not expected to be present in journal databases

40 articles with data only on other risk factors

18 articles with data not stratified by gender

9 articles with smoking prevalences with other criteria

1 insufficient information to characterize population

30 studies** with data on current smoking stratified by gender

30 studies* in five types of population (general population, occupational groups, university students, primary health care users and volunteers)

**14 studies**

general population

**6 studies** primary health care users

**6 studies** university students

**3 studies** occupational groups

**1 study** volunteers

* *E. g.* Eurobarometer study, or other national surveys, whose results are usually published as reports;

** Includes 2 studies obtained directly from the authors.

**Additional file 2.** Main characteristics and results of the studies included in the systematic review and respective prevalence estimates.

| **1^st^ Author, year of publication** | **Year/period of data collection** | **Population type** | **Sampling process** | **Recruitment place** | **Gender** | **Age range**  **Mean (SD)** | **Sample**  **size** | **Current smoker (%)** | **Current smoker criteria** |
| --- | --- | --- | --- | --- | --- | --- | --- | --- | --- |
| dos Reis, 1990 [1] | 1989 * | Primary health care centers users | Probability | Algés | F | 21-40  41-60  61-80 ˠ | 136  178  317 | 24  4  0 | Smoke regularly |
|  |  |  |  |  | M | 21-40  41-60  61-80 ˠ | 87  133  136 | 44  39  21 | Smoke regularly |
| Schlettwein-Gsell, 1991 [2] | 1988-1989 | General population | Probability | Vila Franca de Xira | F | 70-75  ND (ND) | 111 | 1 | Currently smoke |
|  |  |  |  |  | M | 70-75  ND (ND) | 111 | 13 | Currently smoke |
| Steptoe, 1992 [3] | 1990 | University students | Not probability | Lisboa | F | 17-30  22.1 (2.8) | 524 | 34.4 | Daily (at least one cigarette per day) |
|  |  |  |  |  | M |  | 332 | 35.2 | Daily (at least one cigarette per day) |
| da Costa, 1997 [4] | 1994 | Occupational group | Not probability | North region of Portugal | F | ND  32.1 (ND) | 1704 | 3.1 | Currently smoke or had ceased at less than one year |
|  |  |  |  |  | M | ND  36.4 (ND) | 1825 | 21.6 | Currently smoke or had ceased at less than one year |
| Nunes, 1997 [5] | 1995 | Volunteers | Not probability | Viseu | F | 20-29  30-39  40-49  50-59  60-69  70-79  80-89 ˠ | 1173 | 17.3  16.5  4.6  0.3  0.4  0.8  0 | Current smokers |
|  |  |  |  |  | M | 20-29  30-39  40-49  50-59  60-69  70-79  80-89 ˠ | 679 | 35.0  33.3  19.7  13.4  7.7  10.4  12.5 | Current smokers |
| **1^st^ Author, year of publication** | **Year/**  **period of data collection** | **Population type** | **Sampling process** | **Recruitment place** | **Gender** | **Age range**  **Mean (SD)** | **Sample**  **size** | **Current smoker**  **(%)** | **Current smoker criteria** |
| Canhão, 1999 [6] | 1987-1998 | Primary health care centers users | Probability | Lisboa | F | 35-75 ᵼ  ND (ND) | 150 | 6.6 | Current smokers |
|  |  |  |  |  | M | 35-75 ᵼ  ND (ND) | 256 | 28 | Current smokers |
| Simões, 2000 [7] | 1998-1999 | General population | Not probability | Góis | F | 25-29  30-34  35-39  40-44 | 49  42  53  30 | 12.2  7.1  3.7  10.0 | Currently smoke |
|  |  |  |  |  | M | 25-29  30-34  35-39  40-44 | 44  44  48  30 | 38.6  29.5  37.5  36.6 | Currently smoke |
| Torres, 2000 [8] | 1999 * | General population | Probability | Câmara de Lobos | M | 25-65  41.4 (11) | 50 | 62 | Current smokers |
|  |  |  |  | Curral das Freiras | M | 25-65  48.2 (11.3) | 37 | 45 | Current smokers |
| Correia, 2001 [9]** | 2000 | Primary health care centers users | Not probability | North of Portugal | M | 33-73  51 (11) | 40 | 30 | Regular smokers |
| Marques-Vidal, 2001 [10] | 1994-1995 | University students | Probability | Monte da Caparica | F | 17-25  19 (3) | 585 | 17 | Currently smoke |
|  |  |  |  |  | M | 17-25  19.6 (3.6) | 416 | 24 | Currently smoke |
| Steptoe, 2002 [11] | 1999-2001 | University students | Probability | Portugal ‖ | F | 17-30  ND (ND) | 951 | 42.5 | Daily/less than 1 cigarette per day/smoke pipe or cigars |
|  |  |  |  |  | M |  |  | 47.4 | Daily/less than 1 cigarette per day/smoke pipe or cigars |
| **1^st^ Author, year of publication** | **Year/**  **period of data collection** | **Population type** | **Sampling process** | **Recruitment place** | **Gender** | **Age range**  **Mean (SD)** | **Sample**  **size** | **Current smoker**  **(%)** | **Current smoker criteria** |
| Santos, 2004 [12] ¥ | 1999-2003 | General population | Probability | Porto | F | 18-24  25-34  35-44  45-54  55-64  64-74  75-93 | 67  123  234  349  302  253  68 | 40.7  36.2  31.4  15.7  5.8  0.8  1.3 | Current smokers |
|  |  |  |  |  | M | 18-24  25-34  35-44  45-54  55-64  64-74  75-93 | 59  71  146  203  182  158  62 | 44.0  61.5  49.7  38.0  23.8  17.1  11.1 | Current smokers |
| Clemente, 2004 [13] | 2003 * | University students | Not probability | Porto | F | ND  21.3 (2.4) | 228 | 16.4 | Daily (at least one cigarette per day) |
|  |  |  |  |  | M | ND  20.8 (2.2) | 152 | 10.4 | Daily (at least one cigarette per day) |
| Huisman, 2005 [14] | 1998 | General population | Probability | Portugal | F | 25-44  45-64  65-84 ˠ | 1800  1783  1469 | 12  3  0 | Daily (at least one cigarette per day) |
|  |  |  |  |  | M | 25-44  45-64  65-84 ˠ | 1851  1505  1108 | 42  29  13 | Daily (at least one cigarette per day) |
| Almeida, 2007 [15] | 2005-2006 | Occupational group | Not probability | Portugal ‖ | M | 21-ND  37.5 (10.9) | 209 | 42.9 | Smoking daily for at least six months |
| Correia, 2007 [16] § | 2003 | General population | Probability | Lisboa | F | 16-24  25-34  35-44 | 424 | 42  30  23 | Currently smoke |
| Borges, 2008 [17] | 2007 | University students | Not probability | Portugal **** | F | ND  24.24 (1.5) | 161 | 14.9 | Currently smoke |
|  |  |  |  |  | M |  | 94 | 23.4 | Currently smoke |
| **1^st^ Author, year of publication** | **Year/**  **period of data collection** | **Population type** | **Sampling process** | **Recruitment place** | **Gender** | **Age range**  **Mean (SD)** | **Sample**  **size** | **Current smoker**  **(%)** | **Current smoker criteria** |
| Teles, 2008 [18] | 2004-2005 | Primary health care users | Not probability | Portugal | M | 40-69  ND (ND) | 3067 | 34.7 ¶ | Current smokers |
| Sallmen, 2008 [19] | 1997 | Occupational group | Probability | Gaia | F | 15-39  ND (ND) | 406 | 11.58 □ | Daily (at least one cigarette per day) |
| Saleiro, 2008 [20] | 2007 * | University students ***** | Probability | Porto | F | 17-41  ND (ND) | 165 | 10.9 | Smoking daily for at least 6 months |
|  |  |  |  |  | M |  | 173 | 31.8 | Smoking daily for at least 6 months |
| Carmo Martins, 2008 [21] | 2007 * | Primary health care centers users | Not probability | Lisboa | F | 20-30  30-80 | 408 | 25.4  14.4 | Daily (at least one cigarette per day) |
|  |  |  |  |  | M | 20-30  30-80 | 264 | 51.1  26.3 | Daily (at least one cigarette per day) |
| Santos, 2008 [22] | 2004 | General population | Not probability | Açores | F | 18-65  37.78 (9.45) | 5732 | 18.5 | Daily/less than 1 cigarette per day |
|  |  |  |  |  | M |  | 4268 | 42.5 | Daily/less than 1 cigarette per day |
| Cardoso, 2008 [23] | 2008 | General population | Probability | Mainland Portugal | F | 15-84 ˠ  ND (ND) | 3269 | 9.5 | Current smokers |
|  |  |  |  |  | M | 15-84 ˠ  ND (ND) | 3039 | 24.5 | Current smokers |
| Precioso, 2009 [24] | 1987 | General population | Probability | Mainland Portugal | F | 15-24  25-34  35-44  45-54  55-64  65-74  75-84  85-94 ˠ | 3100  2326  2682  2870  2886  2135  1611 | 10.3  12.3  6.3  2.4  1.0  0.5  0.2  0.3 | Currently smoke |
| **1^st^ Author, year of publication** | **Year/**  **period of data collection** | **Population type** | **Sampling process** | **Recruitment place** | **Gender** | **Age range**  **Mean (SD)** | **Sample**  **size** | **Current smoker**  **(%)** | **Current smoker criteria** |
| Precioso, 2009 [24] | 1987 | General population | Probability | Mainland Portugal | M | 15-24  25-34  35-44  45-54  55-64  65-74  75-84  85-94 ˠ | 3367  2279  2303  2522  2471  1768  974 | 30.7  53.0  41.3  32.3  28.1  20.1  17.7  15.9 | Currently smoke |
| Precioso, 2009 [24] | 1995-1996 | General population | Probability | Mainland  Portugal | F | 18-24  25-34  35-44  45-54  55-64  65-74  75-84 ˠ | 2449  2797  3424  3393  3588  3141  2253 | 13.7  17.2  11.2  4.1  1.1  0.6  0.5 | Currently smoke |
|  |  |  |  |  | M | 18-24  25-34  35-44  45-54  55-64  65-74  75-84 ˠ | 2693  2782  3061  3092  3077  2639  1498 | 34.3  48.7  41.8  31.6  21.2  15.5  8.9 | Currently smoke |
| Precioso, 2009 [24] | 1998-1999 | General population | Probability | Mainland  Portugal | F | 15-24  25-34  35-44  45-54  55-64  65-74  75-84  85-94 ˠ | 3124  2908  3367  3407  3398  3218  1876  513 | 10.5  19.6  15.0  6.1  2.2  0.7  0.6  0.2 | Currently smoke |
|  |  |  |  |  | M | 15-24  25-34  35-44  45-54  55-64  65-74  75-84  85-94 ˠ | 3519  2926  3056  3086  2852  2636  1305  252 | 25.8  47.1  45.8  31.6  22.0  14.6  8.0  5.2 | Currently smoke |
| **1^st^ Author, year of publication** | **Year/**  **period of data collection** | **Population type** | **Sampling process** | **Recruitment place** | **Gender** | **Age range**  **Mean (SD)** | **Sample**  **size** | **Current smoker**  **(%)** | **Current smoker criteria** |
| INE, 2009 [25] ¥ | 2005-2006 | General population | Probability | Portugal | F | 15-24  25-34  35-44  45-54  55-64  65-74  75-84  85-94 ˠ | ND  1644  2390  2454  2340  2415  1341  227 | 14.0  16.0  19.1  11.1  5.0  1.4  0.2  0.0 | Currently smoke |
|  |  |  |  |  | M | 15-24  25-34  35-44  45-54  55-64  65-74  75-84  85-94 ˠ | ND  1077  1541  1710  1623  1681  996  130 | 26.0  34.5  41.3  31.0  19.5  12.4  6.8  0.6 | Currently smoke |
| Correia, 2009 [26] ¥ | 2008 | General population | Probability | Mainland  Portugal | F | 40-49  50-59  60-69  70-79  80-89 | 247  314  399  362  153 | 16.3  9.1  3.1  2.2  0 | Daily (at least one cigarette per day) |
|  |  |  |  |  | M | 40-49  50-59  60-69  70-79  80-89 | 76  106  108  111  46 | 30.3  26.8  7.9  7.3  0 | Daily (at least one cigarette per day) |
| Lobão, 2010 [27] | 2007 | Primary health care centers users | Not probability | Vila Nova de Gaia | F | 18-84 ˠ  ND (ND) | 337 | 19 | Currently smoke |
|  |  |  |  |  | M | 18-84 ˠ  ND (ND) | 165 | 25 | Currently smoke |
| Eurobarometer, 2012 [28] | 2012 | General population | Probability | Portugal | F | 15-84 ˠ  ND (ND) | 1000 | 24 | Currently smoke |
|  |  |  |  |  | M | 15-84 ˠ  ND (ND) |  | 32 | Currently smoke |

ˠ For surveys that did not report the age range of the participants, but reported data by age groups, we considered the upper/lower limit by assuming the same width for extreme classes as that of the closest class (*e.g.* for surveys reporting data in participants aged <30, 30–39, 40–49, and ≥50 years, we considered the overall range as 20–59 years);

* When the period of data collection was not reported we assumed the publication year minus the median difference between the publication year and date of data collection in the articles for which that information was available (1.0 years);

ᵼ Assumed the same age range of the cases, since the controls were matched by sex and age (Canhao et al., 1999);

** Only data from the controls of the study was considered (Correia et al., 2001);

‖ Data from a Portuguese region not further specified;

¥ Age- and sex- estimates obtained directly from the authors;

§ Data on the prevalence of smoking only was considered for the period prior to pregnancy (Correia et al., 2007);

**** Data from all de medical faculties of Portugal (Borges et al., 2008);

¶ Prevalence of current smokers was computed by subtracting to 100% the prevalence of never and ex smokers (Teles et al., 2008);

□ Weighted mean (Sallmen et al., 2008);

***** Data from medical students (Borges et al., 2008; Saleiro et al., 2008);

SD – standard deviation;

ND – no data.

**References**

1. dos Reis RP, dos Reis HP: **[Cardiovascular risk factors in the list of patients of a general practitioner].** *Rev Port Cardiol* 1990, **9:**607-612.

2. Schlettwein-Gsell D, de Prins L, Ferry M: **Life-style: marital status, education, living situation, social contacts, personal habits (smoking, drinking). Euronut SENECA investigators.** *Eur J Clin Nutr* 1991, **45 Suppl 3:**153-168.

3. Steptoe A, Wardle J: **Cognitive predictors of health behaviour in contrasting regions of Europe.** *Br J Clin Psychol* 1992, **31 ( Pt 4):**485-502.

4. da Costa JT, Barros H, Macedo JA, Ribeiro H, Mayan O, Pinto AS: **[Respiratory symptoms in the textile industry. Their prevalence in the Vale do Ave].** *Acta Med Port* 1997, **10:**7-14.

5. Nunes L, Pipa J, Nascimento C, Costa A, Cabral C, Almeida L, Soares N, Ferreira J, Portugal A, Veiga L, et al: **[Prevalence of several cardiovascular risk factors in a population in the municipality of Viseu].** *Rev Port Cardiol* 1997, **16:**703-707, 664.

6. Canhao P, Falcao F, Pinho e Melo T, Ferro H, Ferro J: **Vascular risk factors for perimesencephalic nonaneurysmal subarachnoid hemorrhage.** *J Neurol* 1999, **246:**492-496.

7. Simoes JA, Gama ME, Contente CB: **Prevalence of cardiovascular risk factors in a rural population between 25 and 44 years old.** *Rev Port Cardiol* 2000, **19:**693-703.

8. Torres IC, Mira L, Ornelas CP, Melim A: **Study of the effects of dietary fish intake on serum lipids and lipoproteins in two populations with different dietary habits.** *Br J Nutr* 2000, **83:**371-379.

9. Correia AM, Goncalves G, Reis J, Cruz JM, Castro e Freitas JA: **An outbreak of legionnaires' disease in a municipality in northern Portugal.** *Euro Surveill* 2001, **6:**121-124.

10. Marques-Vidal P, Llobet S, Carvalho Rodrigues JA, Halpern MJ: **Cardiovascular risk factor levels in Portuguese students.** *Acta Cardiol* 2001, **56:**97-101.

11. Steptoe A, Wardle J, Cui W, Bellisle F, Zotti AM, Baranyai R, Sanderman R: **Trends in smoking, diet, physical exercise, and attitudes toward health in European university students from 13 countries, 1990-2000.** *Prev Med* 2002, **35:**97-104.

12. Santos AC, Barros H: **Smoking patterns in a community sample of Portuguese adults, 1999-2000.** *Prev Med* 2004, **38:**114-119.

13. Clemente L, Moreira P, Oliveira B, Almeida MD: **[Body mass index: sensitivity and specificity].** *Acta Med Port* 2004, **17:**353-358.

14. Huisman M, Kunst AE, Mackenbach JP: **Educational inequalities in smoking among men and women aged 16 years and older in 11 European countries.** *Tob Control* 2005, **14:**106-113.

15. Almeida AG, Duarte R, Mieiro L, Paiva AC, Rodrigues AM, Almeida MH, Barbara C: **[Pulmonary function in Portuguese firefighters].** *Rev Port Pneumol* 2007, **13:**349-364.

16. Correia S, Nascimento C, Gouveia R, Martins S, Sandes AR, Figueira J, Valente S, Rocha E, Da Silva L: **[Pregnancy and smoking: an opportunity to change behaviours].** *Acta Med Port* 2007, **20:**201-207.

17. Borges A, Marques F, Lima J, Costa L, Goncalves P, Fernandes R, Goncalves N: **Smoking habits of sixth year medical students and anti-smoking measures in Portugal.** *Rev Port Pneumol* 2008, **14:**379-390.

18. Teles AG, Carreira M, Alarcao V, Sociol D, Aragues JM, Lopes L, Mascarenhas M, Costa JG: **Prevalence, severity, and risk factors for erectile dysfunction in a representative sample of 3,548 portuguese men aged 40 to 69 years attending primary healthcare centers: results of the Portuguese erectile dysfunction study.** *J Sex Med* 2008, **5:**1317-1324.

19. Sallmen M, Neto M, Mayan ON: **Reduced fertility among shoe manufacturing workers.** *Occup Environ Med* 2008, **65:**518-524.

20. Saleiro S, Damas C, Gomes I: **Smoking habits and awareness of smoking risks depending on academic background in university students.** *Rev Port Pneumol* 2008, **14:**231-238.

21. Carmo Martins M, Lima Faleiro L, Rodrigues MO, Albergaria I, Fonseca A: **[Influence of the APOE genotypes in some atherosclerotic risk factors].** *Acta Med Port* 2008, **21:**433-440.

22. Santos R, Aires L, Santos P, Ribeiro JC, Mota J: **Prevalence of overweight and obesity in a Portuguese sample of adults: results from the Azorean Physical Activity and Health Study.** *Am J Hum Biol* 2008, **20:**78-85.

23. Cardoso C, Plantier T. 2008. Acompanhamento estatístico e epidemiológico do consumo de tabaco em Portugal. Estudo do impacte da Lei n.º 37/2007, de 14 de Agosto. Available at: <http://www.dgs.pt/>

24. Precioso J, Calheiros J, Pereira D, Campos H, Antunes H, Rebelo L, Bonito J: **[Prevalence and smoking trends in Portugal and Europe].** *Acta Med Port* 2009, **22:**335-348.

25. Instituto Nacional de Estatística, Instituto Nacional de Saúde Ricardo Jorge: **Inquérito Nacional de Saúde, 2005-2006.** 2009.

26. Correia S, Dinis P, Rolo F, Lunet N: **Prevalence, treatment and known risk factors of urinary incontinence and overactive bladder in the non-institutionalized Portuguese population.** *Int Urogynecol J Pelvic Floor Dysfunct* 2009, **20:**1481-1489.

27. Lobao A, Marques P, Leite C, Almeida M, Araujo P, Cardoso V, Pinto ME, Vidal F: **[Smoking and cardiovascular risk factors in Barao do Corvo Health Center].** *Acta Med Port* 2010, **23:**159-166.

28. European Comission: *Attitudes of Europeans Towards Tobacco. Special Eurobarometer 385 / Wave EB77.1.* 2012.
